# Development of a patient-reported outcome measure for gastrointestinal recovery after surgery (PRO-diGI)

**DOI:** 10.1093/bjs/znaf055

**Published:** 2025-04-10

**Authors:** Matthew J Lee, Daniel M Baker, Debby Hawkins, Sue Blackwell, Robert Arnott, Deena Harji, Gabrielle Thorpe, Stephen J Chapman, Georgina L Jones, the PRO-diGI Collaborators, Ewen Griffiths, Rebecca Hancox, Arlo Whitehouse, Michelle Bates, Claire McNeill, Manijeh Ghods, Andrew McDarby, Andrew Shepperson, Clare Hutton, Syed Rahman-Casana, Daniel Ashmore, Georgia Hooton, Rebecca Pugh, Timothy Wilson, Victoria Allinson, John Liam O'Hare, Bernadette Collinson, Karen Convery, James Clark, Charlotte Barker-Kirby, Eve Fletcher, Suzanne Dean, Eleanor Walker, Frank McDermott, Linda Park, Melissa-Rose Bennett, Sophie Ashman, Sammy Conroy, Caroline Steele, Charmaine Shovelton, Kate James, Eric Mbogu, Karen Roberts, Lucia Sharp, Lynne Palmer, Amy Smith, Fiona Wakinshaw, Jennifer Henderson, Madeleine Richardson, Cleo Kenington, Claire Gilmartin, Hong Ju, Mercedes Lucas Mejia

**Affiliations:** Department of Applied Health Sciences, College of Medical and Dental Sciences, University of Birmingham, Birmingham, UK; Division of Clinical Medicine, Sheffield Medical School, University of Sheffield, Sheffield, UK; Department of Surgery, Leeds Teaching Hospitals NHS Foundation Trust, Leeds, UK; Academic Directorate of General Surgery, Sheffield Teaching Hospitals NHS Foundation Trust, Sheffield, UK; Lay representative, Birmingham, UK; Lay representative, Birmingham, UK; Department of Colorectal Surgery, Manchester Foundation NHS Trust, Manchester, UK; School of Health Sciences, University of East Anglia, Norwich, UK; Leeds Institute of Medical Research at St James’s, University of Leeds, Leeds, UK; Department of Health Psychology, School of Humanities and Social Sciences, Leeds Beckett University, Leeds, UK

## Abstract

**Background:**

After major abdominal surgery, patients may experience significant gastrointestinal dysfunction, including postoperative ileus. Many clinical tools are used to measure this dysfunction, but there is no patient-reported outcome measure (PROM) specific to this group. The aim of this study was to develop a new PROM for this common condition.

**Methods:**

A four-stage approach was undertaken. Stage 1 used semi-structured interviews with 29 patients to explore experiences of gastrointestinal recovery and develop a draft questionnaire. Stage 2 solicited feedback from 18 patients and 15 clinical experts on the face validity of the proposed tool using the Questionnaire on Questionnaires (QQ-10). Stage 3 recruited 297 patients to complete the questionnaire. Principal component analysis reduced the items and identified the domain structure. Test-retest reliability and a pilot assessment of responsiveness were assessed in stage 4 in a sample of 100 patients and in a sample of 68 patients respectively.

**Results:**

The interviews generated 26 subthemes across gastrointestinal recovery and general well-being. An initial questionnaire containing 44 items was developed. The QQ-10 demonstrated high value and low burden, supporting face validity. Tests to reduce the items and identify the domain structure resulted in a 15-item questionnaire across four domains (nausea, eating, well-being, and bowels). Test-retest reliability showed intraclass correlation coefficient values ≥0.7 for all domains. Pilot responsiveness was demonstrated through differences in pre- and post-surgical scores.

**Conclusion:**

PRO-diGI is a PROM for gastrointestinal dysfunction after major abdominal surgery that shows good psychometric properties and demonstrates face validity, reliability, and responsiveness. This now needs external validation to facilitate broader implementation.

## Teaser

This study describes a four-stage development of a patient-reported outcome measure for gastrointestinal recovery. This results in a tool addressing four key domains: nausea, eating, well-being, and bowels.

## Introduction

Millions of patients undergo major abdominal surgery worldwide each year^1^. After such operations, 30% of patients may experience an interval of significant gastrointestinal dysfunction^2^. This manifests as nausea, vomiting, obstipation, and abdominal pain. This can lead to loss of intestinal function for a short interval of time, commonly termed ‘postoperative ileus’. This is associated with delayed recovery and is associated with an increase in healthcare costs of around 66% and an increase in hospital length of stay of around 5 days^3,4^. Patients may also report symptoms related to altered gastrointestinal function for several weeks after surgery^5^. This condition has been identified as a research priority by patients and clinicians^6^.

Previous work has demonstrated a range of clinical and radiological measures to assess for return of gastrointestinal function^7^. However, many of these assess a single dimension of recovery in a binary manner, at a single point in time; for example passage of flatus at day 3 post-surgery^8^. A recently agreed international consensus-based core outcome set recommended the use of a condition-specific patient-reported outcome measure (PROM) to be implemented in studies of gastrointestinal recovery^9^. Whilst tools for measuring gastrointestinal-related quality of life exist, they are designed for chronic conditions in outpatient settings^10,11^.

The aim of this study was to address this gap by developing a PROM to assess gastrointestinal recovery.

## Methods

### Overview

PRO-diGI followed a four-stage development process. Stage 1 used an exploratory qualitative approach to generate a longlist of candidate items for inclusion in the PROM. Stage 2 undertook an assessment of face validity of the proposed tool. Stage 3 used classical test construction, and tests of internal-consistency and reliability to identify the domain structure and reduce the items. Stage 4 assessed test-retest reliability, responsiveness, and known-group differences. The study was registered prospectively at clinicaltrials.gov (NCT05315765) and received approval from the NHS Wales Research Ethics Committee 6 (21/WA/0231). It is reported with reference to the Guidance for Reporting Involvement of Patients and the Public Short Form (GRIPP-2 SF)^12^ framework. The *Supplementary material* maps the study to the COnsensus-based Standards for the selection of health Measurement INstruments (COSMIN) taxonomy of measurement properties^13^.

### Study steering group and patient involvement

Members of the steering group included patient representatives with relevant lived experiences, who provided patient and public involvement (PPI). Research professionals included health psychologists, colorectal nursing experts, and surgeons with relevant clinical and academic experience. Patient representatives contributed to all stages of the study, including concept, design, analysis, and preparation of outputs.

### Inclusion and exclusion criteria

Patients undergoing major emergency laparotomy^14^ or planned major abdominal surgery (for example resectional surgery for cancer or inflammatory bowel disease), major gynaecological surgery (for example transabdominal hysterectomy), or major urological surgery (for example cystectomy) were eligible to participate. As the survey was developed in English, patients needed to be able to converse in English to participate. Additional selection criteria were applied in stage 4.

### Stage 1: item generation

Qualitative interviews were undertaken to develop the items and domains for the new PROM. Question coverage was informed by mapping the domains from commonly used tools for assessment of chronic gastrointestinal health (Gastrointestinal Symptom Rating Scale [GSRS]^11^ and Gastrointestinal Quality of Life Index [GI-QLI]^10^). A semi-structured interview schedule was developed by the research team with input from PPI representatives.

Participants were recruited from five UK hospitals according to the eligibility criteria. Potential participants were approached before discharge and invited to participate. If informed consent was secured, a virtual or telephone interview was arranged at a convenient time within 2–4 weeks after hospital discharge. This flexibility was intentional to allow capture of experiences during the early recovery journey. A brief follow-up telephone call was undertaken 6 weeks later to identify any further experiences related to recovery. Recruitment was intended to provide a range of participants with regard to age, sex, parent specialty, and ethnicity.

Interviews were performed by two non-clinical researchers, who were experienced in undertaking qualitative interview-based research. The interviewers were both male and had no clinical background. Interviews were recorded and transcribed verbatim. Transcripts were iteratively coded by two members of the team using a content analysis approach^15^. The researchers independently coded five interviews each before agreeing on a final framework. This was reviewed by the wider team, including PPI representatives. Interviews were continued until thematic saturation was achieved. Coding was performed using NBibo 11 (QSR international, Melbourne Australia). A full summary of the approach used has been published previously^5^.

Following stage 1, candidate items were used to develop a draft questionnaire. This draft questionnaire and the wording of questions was reviewed by the steering group, including PPI representatives. It also included a global anchor question on a 100 mm scale where participants could provide an overall rating of their gastrointestinal function on a 0–100 scale.

### Stage 2: face validity assessment

Face validity was assessed by patients (eligibility as per stage 1). Clinicians involved in the care of postoperative patients, or with research interests in gastrointestinal recovery, and who were English speaking were also invited to participate. Feedback was sought from researchers and clinicians in the UK and from anglophone countries. Participants were provided with a copy of the draft questionnaire and asked to provide structured feedback using the Questionnaire on Questionnaires (QQ-10), a validated measure for assessing the face validity of questionnaires^16^. This includes ten questions assessing the acceptability of the measure to users. Free-text comments on phrasing, missing items, and over-represented items were also recorded. The QQ-10 was scored by converting the five-point Likert scale ranging from ‘strongly disagree’ to ‘strongly agree’ to a point value ranging from zero to four. A score for the ‘value’ domain was calculated by summing the first six questions and a score for the ‘burden’ domain was calculated by summing the last four questions^16^. The maximum scores for each domain are 24 and 16 respectively. Higher ‘value’ domain scores and lower ‘burden’ domain scores are preferred.

The median and interquartile range (i.q.r.) were calculated for each question and domain score. Free-text feedback was reviewed by the study team and appropriate edits made to the questionnaire. The recruitment target was 15–20 patients and the same number of clinicians. Feedback from this stage was used to refine the questionnaire before progression to stage 3.

### Stage 3: domain development and item reduction

The draft questionnaire underwent further refinement through large-scale completion at ten hospital sites. Participant eligibility was as outlined in stage 1. Participants were invited to participate when they felt able after surgery, but before hospital discharge. Basic demographic data collected included age, sex, operation type, and whether the participant had met the validated composite outcome for gastrointestinal recovery (gastrointestinal-2 (GI-2) outcome)^8^ at the time of completion.

Data were analysed using principal component analysis (PCA) with varimax rotation. This is an analytical approach related to factor analysis, allowing the identification of related items in a questionnaire^17^. It facilitates the identification of ‘components’ or groupings of items and can help to identify redundant items. The minimum sample size for this is accepted as five times the number of items in the questionnaire being assessed^18^. Appropriateness of sample size can be determined through assessment of the Kaiser-Meyer-Olkin value (>0.8 is considered acceptable) and a significant Bartlett’s test (*P* < 0.050). PCA was run iteratively to determine the most psychometrically robust structure of the questionnaire. To extract the factors, corresponding eigenvalues >1, scree plots, and minimum factor loadings of 0.40 were selected.

Cronbach’s alpha and omega values were calculated to assess the internal consistency of each construct, with values of ≥0.7 considered acceptable. Item–total correlations and the mean inter-item correlations were also calculated. Minimum correlation coefficients of >0.40 and >0.30 were adopted respectively^19^. Where a component did not meet either of these thresholds, it was removed and the analysis rerun.

### Stage 4: assessment of reliability and responsiveness

The refined questionnaire developed in stage 3 was used in this stage.

#### Stage 4a: test-retest reliability

Test-retest reliability was assessed by two completions of the tool at a point when the patients’ responses could be considered stable. Based on stage 1, it was determined that recovery is an ongoing process for several weeks and therefore stability was best measured at a plateau in recovery. Patients were therefore recruited to complete the reduced-item questionnaire twice on their day of hospital discharge, with an interval of no less than 4 h between administrations.

Basic demographic and operative data were also captured. Scores for the two time points were calculated. Stability was demonstrated using Wilcoxon’s rank-sum and the intraclass correlation coefficient (ICC; two-way mixed-effect ANOVA model with interaction for absolute agreement between single scores) for each scoring domain on patients with exact matches in their anchor scores. An ICC value of ≥0.7 is considered acceptable^20^. A further assessment using the QQ-10 was undertaken.

To calculate PRO-diGI scores, each item was allocated a value according to the five-point Likert scale used, with the first response option scoring four and the last response option scoring zero. Scores for each domain were converted into percentages by calculating the sum of scores for each domain and dividing by the maximum score for the domain where all responses were offered, then multiplying by 100. Therefore, a higher score is associated with better gastrointestinal recovery. No domain score was calculated where a constituent item was missing. Scores were compared for those patients with the same overall gastrointestinal function rating, indicating stability. A sample size with >50 complete data sets was considered adequate to assess this^21^.

#### Stage 4b: pilot responsiveness assessment

A further key assessment is the ability of a tool to detect a difference between two different states. Patients undergoing elective major abdominal surgery (as defined in stage 1) were invited to complete the questionnaire before operation on the day of surgery and then again at day 3 post-surgery. Basic demographic and operative data were also collected. Responsiveness was assessed using distribution-based methods on the whole group. No patient-reported anchor was used in this stage. Three approaches using the retest data were used: the difference between test and retest mean scores by PRO-diGI domain using Cohen’s D; calculating a 0.5 s.d. unit at baseline for each PRO-diGI domain; and estimating the responder definition threshold as one standard error of measurement.

A sample size with >50 complete data sets was considered adequate to assess this^21^.

#### Stage 4c: construct validity (known-group comparisons)

Known-group validity refers to whether a tool can discriminate between two groups known to differ on the variable of interest^22^. This provides assurance that the tool behaves as expected. This was performed by comparing median domain scores between two groups with characteristics of interest. The *a priori* hypotheses developed at study commencement were: patients who have minimally invasive surgery will demonstrate better PRO-diGI scores on day 3 assessment than those who have open surgery^23^; patients who have met the GI-2 outcome will demonstrate better PRO-diGI scores on day 3 assessment than those who have not met the outcome^8^; and patients who have emergency surgery will demonstrate worse PRO-diGI scores on day 3 assessment than those who have elective surgery^23,24^.

## Results

### Stage 1: item generation

A total of 43 participants were consented between October 2021 and January 2022, and 29 interviews were completed. Of the participants who did not complete interviews, one no longer had the capacity to consent, one declined participation, and the remainder did not respond to telephone contacts to arrange interviews. The characteristics of participants are presented in *Table 1*. Data saturation was achieved after 25 interviews, with a further 4 interviews demonstrating no new themes. Interviews were completed at a median of 22 (range 9–58) days after surgery. The median interview length was 32 (range 20–71) min.

**Table 1:** Participants in study

Characteristic	Stage 1 (*n* = 29)	Stage 2 (*n* = 18)	Stage 3 (*n* = 297)	Stage 4a (*n* = 100)	Stage 4b (*n* = 68)
**Sex**					
Male	12 (42)	10 (56)	131 (44.1)	44 (44.0)	36 (53)
Female	17 (58)	8 (44)	166 (55.9)	54 (54.0)	32 (47)
Not stated				2 (2.0)	
**Age (years), median (interquartile range)**	64 (50–73)	66 (53–72)	63 (53–73)	66 (55–74)	66 (55–72)
**Ethnicity**					
Asian or Asian British	1 (3)	*	6 (2.0)	1 (1.0)	4 (6)
Black British, Caribbean, or African	1 (3)	*	7 (2.4)	3 (3.0)	–
Other ethnic group	0 (0)	*	5 (1.7)	3 (3.0)	1 (1)
White	26 (90)	*	278 (94)	92 (92.0)	63 (93)
Unknown	1 (3)	*	1 (0.3)	–	–
**Type of admission**					
Elective	16 (55)	9 (50)	215 (72.4)	86 (86.0)	67 (99)
Emergency	13 (45)	9 (50)	80 (26.9)	14 (14.0)	1 (1)
Unknown	–	–	2 (0.7)	1 (1.0)	–
**Operation type**					
Conservatively managed SBO	1 (3)	–	8 (2.7)	1 (1.0)	–
Gastrointestinal	20 (69)	14 (78)	242 (81.5)	95 (95.0)	64 (94)
Gynaecological	5 (17)	2 (11)	31 (10.4)	2 (2.0)	1 (1)
Urological	3 (10)	2 (11)	16 (5.4)	2 (2.0)	3 (3)
**Operative approach**					
Robotic	–	–	10 (3.4)	4 (4.0)	2 (3)
Laparoscopic	–	–	136 (45.8)	34 (34.0)	33 (49)
Minimally invasive (not specified)	9 (31)	–	–	–	–
Open	17 (59)	–	133 (44.8)	56 (56.0)	30 (44)
NA	3 (10)	–	14 (4.8)	4 (4.0)	3 (4)
**Was a new stoma formed?**					
NA	–	–	42 (14.1)	9 (9.0)	–
No	–	–	164 (55.2)	61 (61.0)	53 (78)
Yes	–	–	88 (29.6)	28 (28.0)	15 (22)

Values are *n* (%) unless otherwise indicated. *Not collected in round 2. SBO, small bowel obstruction; NA, not applicable.

**Fig. 1:**
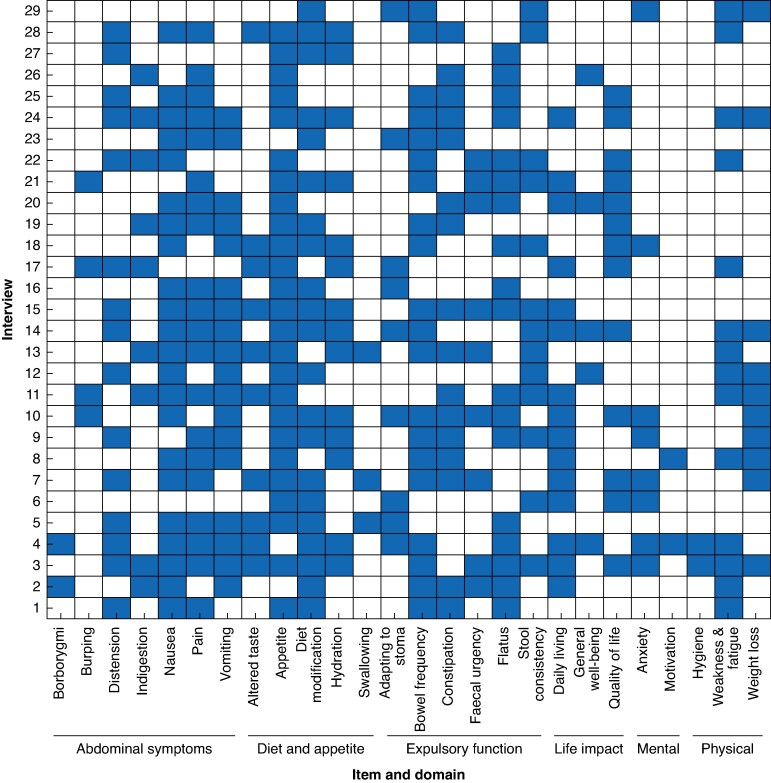
Heatmap of themes reported in interviews

Two overarching themes, with six domains, and a total of 26 subthemes were generated (see *Fig. 1*). ‘General recovery’ included life impact, mental, and physical effects. ‘Gastrointestinal symptoms’ included abdominal symptoms, diet and appetite, and expulsory function. *Figure 1* shows how themes were represented across interviews.

### Stage 2: face validity assessment

Stage 1 led to the development of a 44-item questionnaire. This addressed: flatus (3 items); bowel function (related to defaecation) (10 items); eating and drinking (13 items); nausea and vomiting (5 items); abdominal symptoms (4 items); and general symptoms and well-being (9 items).

The questionnaire also had a single item to rate gastrointestinal function on the day of completion, using a visual analogue scale ranging from 0 (worst function imaginable) to 100 (perfect function).

**Fig. 2:**
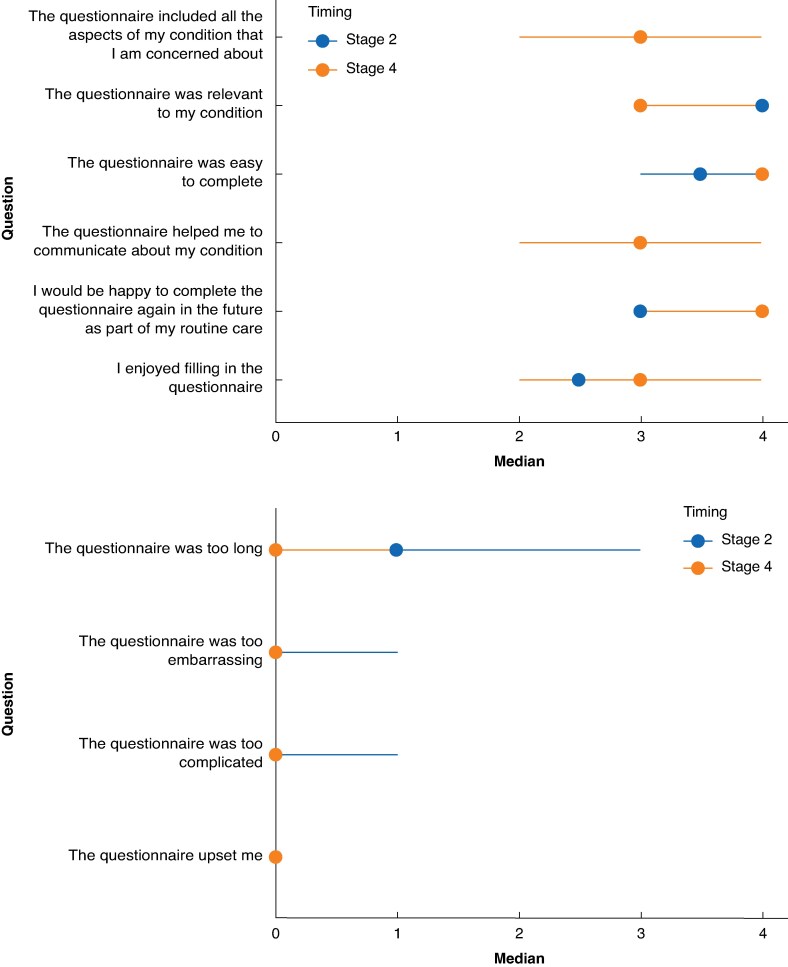
Comparison of QQ-10 scores in stages 2 and 4 QQ-10, Questionnaire on Questionnaires.

Face validity assessment was completed by 18 patients. Of these, 10 were male, 9 were elective (planned) presentations, and 14 were treated for gastrointestinal conditions (*Table 1*). The 15 clinical experts included one urologist, one gynaecologist, two dietitians, and the remaining respondents were surgeons. Experts were drawn from the UK, Ireland, Belgium, New Zealand, and Australia. Quantitative assessment demonstrated positive feedback (*Fig. 2*). There were high median scores for the ‘value’ domains (median of 19 (i.q.r. 17–19.75) and 21 (i.q.r. 18–21.5) for patients and clinicians respectively). There were low median scores for the ‘burden’ domains (median of 1.5 (i.q.r. 0–3) and 3 (i.q.r. 2–4.5) for patients and clinicians respectively), suggesting acceptability and face validity of the tool (*Fig. 2*). Free-text feedback highlighted issues around specific phrases and their interpretation into non-UK English, for example ‘belly’ and ‘sick’. Respondents also indicated that there was a large number of items in this stage, as expected before item reduction.

### Stage 3: domain development and item reduction

A total of 297 participants completed the survey during their hospital admission (*Table 1*).

On initial factor analysis, the Kaiser-Meyer-Olkin value was 0.758 and the Bartlett’s test of sphericity was significant (*P* < 0.001), indicating that the sample size was sufficient. A review of the correlation matrix revealed some collinearity between the items.

Iterative PCA was performed, with Cronbach’s alpha and omega values calculated for each factor. Factors not reaching the 0.7 threshold for either value were removed and the PCA rerun. The PCA was run four times to achieve an acceptable solution. The results of this are shown in *Table 2*. This generated the best solution of four factors across 15 items, with no cross loadings, and all minimum thresholds were met. All item–total correlations and the mean inter-item correlations exceeded 0.40 and 0.30 respectively. An additional exploratory factor analysis using promax rotation was performed and this replicated the final structure.

**Table 2:** Principal component analysis

	Item–total correlations	Mean inter-item correlations (range)	Cronbach’s alpha	Omega
**Factor 1: nausea**		0.470 (0.232–0.806)	0.780	0.755
Have you vomited (been sick) after eating?	0.630			
Have you felt nauseated after eating?	0.741			
Have you vomited (been sick)?	0.517			
Have you felt nauseated?	0.488			
**Factor 2: eating**		0.393 (0.227–0.603)	0.763	0.760
Have you had to change the type of food you eat?	0.523			
Have you had to limit how much food you eat?	0.491			
Has it taken you longer than normal to eat?	0.622			
Have you found it difficult to eat?	0.606			
Have you had difficulty swallowing food?	0.432			
**Factor 3: well-being**		0.567 (0.466–0.733)	0.795	0.806
Do you feel weaker than usual?	0.716			
Have you felt more tired than usual?	0.689			
Has it been difficult to do activities to help you relax?	0.522			
**Factor 4: bowels**		0.631 (0.577–0.702)	0.821	0.823
Has it been difficult to control your bowels/avoid soiling yourself?	0.730			
Have you had to rush to the toilet to open your bowels (do a poo)?	0.701			
Have you had diarrhoea (watery poo)?	0.645			

### Stage 4: assessment of reliability and responsiveness

**Fig. 3:**
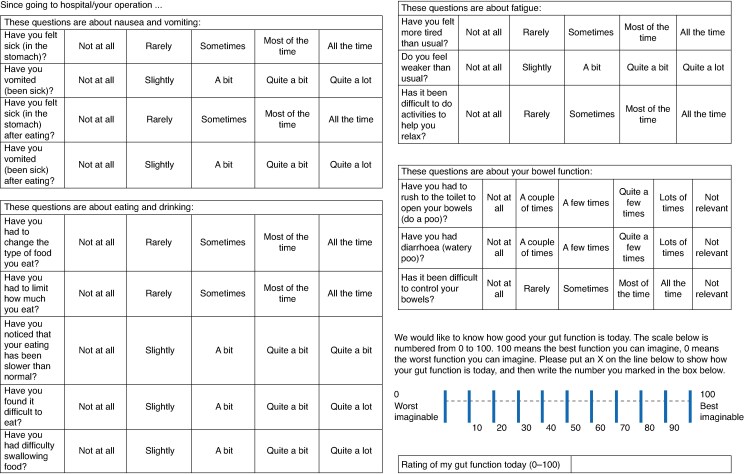
Final version of the PRO-diGI questionnaire The PRO-diGI questionnaire is copyright 2024 by Sheffield Teaching Hospitals. Licensing queries should be directed to Inspired Health Outcomes, Oxford, UK.

Participant characteristics are shown in *Table 1* and the final version of the questionnaire is shown in *Fig. 3*.

#### Stage 4a: test-retest reliability

Test-retest reliability was confirmed, with ICC values of ≥0.7 across each domain (*Table 3*). Additional assessment of face validity by patients using QQ-10 indicated good acceptability (median of 18 (i.q.r. 16–22)) and a low burden of completion (median of 0 (i.q.r. 0–2)). A comparison of patient scores for QQ-10 questions at stages 2 and 4 are presented in *Fig. 2*.

**Table 3:** Test-retest results for those that maintained the same overall rating of gastrointestinal function

PRO-diGI domain	*n*	Test, mean(s.d.)	Retest, mean(s.d.)	*P*	Intraclass correlation coefficient
Nausea	65	78.1 (21.1)	82.5 (19.8)	0.013*	0.70
Eating	65	56.9 (25.6)	59.8 (26.1)	0.122	0.84
Well-being	65	42.2 (27.4)	42.7 (27.8)	0.518	0.92
Bowels	43	73.4 (30.2)	76.9 (29.5)	0.161	0.79

100 = best health and 0 = worst health. *P ≤ 0.050.

#### Stage 4b: pilot responsiveness assessment

**Fig. 4:**
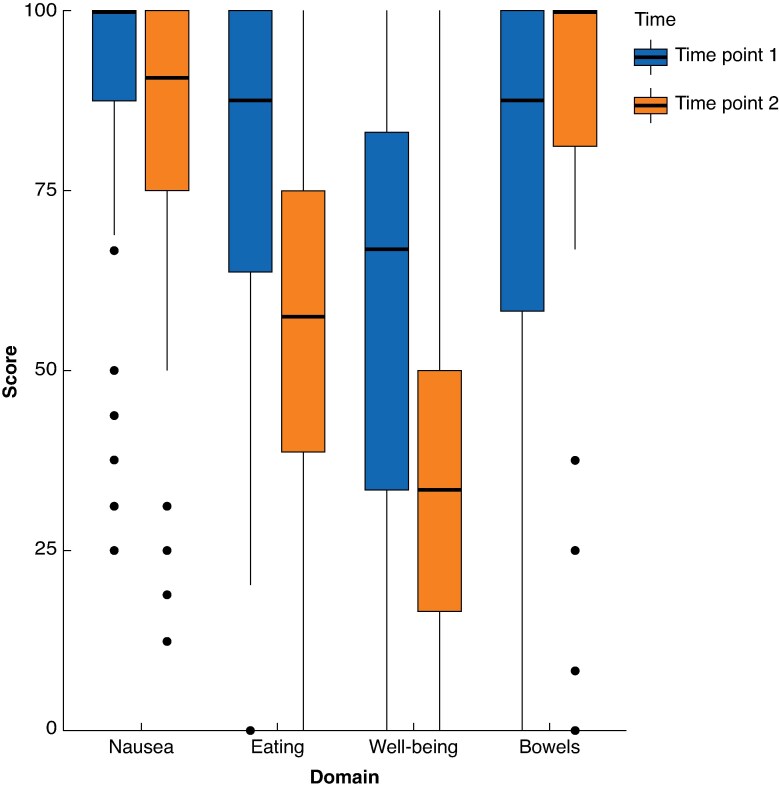
Comparison of pre- and post-surgery PRO-diGI scores Higher scores indicate better function.

Using the distribution method for the whole group, there was a significant difference between preoperative and postoperative measurements in all domains (*Table 4* and *Fig. 4*), with postoperative scores typically lower (worse) than preoperative scores. This is in keeping with gastrointestinal dysfunction. It was notable that postoperative bowel function appears improved after surgery.

**Table 4:** Distribution-based estimates of PRO-diGI domain change scores for the group overall

PRO-diGI domain	*n*	Day of surgery, mean(s.d.)	Day 3 post-surgery, mean (s.d.)	Change, mean(s.d.)	*P*	Effect size (Cohen's D)	Standard error of measurement	Day 1 0.5 s.d.
Nausea	68	89.6 (19.0)	84.3 (20.5)	5.3 (25.2)	0.015*	0.28	7.35	9.5
Eating	68	78.2 (26.0)	53.8 (27.9)	24.4 (36.9)	<0.001*	0.94	12.0	13.0
Well-being	68	61.4 (31.3)	37.1 (26.8)	24.3 (40.0)	<0.001*	0.77	10.9	15.7
Bowels	52	75.4 (28.4)	85.7 (26.5)	−12.3 (33.2)	0.009*	−0.40	11.41	14.2

100 = best health, 0 = worst health. *P ≤ 0.050.

#### Stage 4c: construct validity (known-group comparisons)

In relation to one of the hypotheses for known-group validity, those who had met the GI-2 outcome at day 3 showed a trend towards higher (better) scores compared with those who had not met the outcome; however, this was not significant. The group who had not met the GI-2 outcome scored significantly higher (better) in the bowel domain (*Table 5*). In relation to another hypothesis for known-group validity, a comparison of scores by operative approach demonstrated significantly higher (better) values related to the eating and well-being domains in the minimally invasive group *versus* the open group at day 3 (*Table 5*). The final hypothesis was not tested, as recruitment was focused on elective surgery patients in stage 4b.

**Table 5:** Known-group comparisons

PRO-diGI domain	*n*	GI-2 outcome met, mean(s.d.)	GI-2 outcome not met, mean(s.d.)	*P*	*n*	Open, mean(s.d.)	Minimally invasive, mean(s.d.)	*P*
Nausea	35	85.7 (20.6)	82.5 (20.9)	0.373	65	82.7 (19.9)	80.3 (22.3)	0.620
Eating	35	57.7 (24.9)	48.1 (30.8)	0.162	65	53.4 (28.4)	67.0 (26.1)	0.019*
Well-being	35	39.0 (27.2)	34.7 (26.8)	0.568	65	35.6 (27.4)	51.2 (27.7)	0.006*
Bowels	30	78.1 (31.9)	95.8 (10.3)	0.008*	65	75.0 (28.5)	77.1 (32.9)	0.280

100 = best health, 0 = worst health. *P* values calculated using the Mann–Whitney *U* test. *P ≤ 0.050. GI-2, gastrointestinal-2.

## Discussion

The PRO-diGI PROM is a new tool to measure gastrointestinal recovery after surgery or during recovery after acute surgical conditions. It has been developed with reference to good PROM development practice^25^ and in collaboration with key stakeholders, including patients. It demonstrates a more multidimensional approach to assessment of gastrointestinal recovery than currently favoured measures such as the GI-2 outcome^7^. PRO-diGI demonstrates many of the characteristics essential to be a useful PROM. Specifically, it has demonstrated construct validity, test-retest reliability, and responsiveness. These characteristics will facilitate use in practice and future research.

Current postoperative PROMs exist to assess global and functional recovery^26^. There is a general lack of standardization of measurement of gastrointestinal recovery, contributing substantially to the unmet challenge of measuring and treating conditions such as postoperative ileus and small bowel obstruction^7^. The development of this tool addresses one of the key gaps in the previously developed gastrointestinal core outcome sets^9,27^. The additional value of the PRO-diGI tool is demonstrated through its focus on gastrointestinal symptoms. Previous studies have focused on resumption of expulsory function as a marker of recovery, likely driven by traditional measures considered to be important by clinicians^7^. This study found broader features of bowel function, such as incontinence and frequency, were troublesome symptoms, which are poorly recorded elsewhere. It is notable that bowel function appears improved after surgery. This might reflect issues with missing data in the domain or, more likely, early completion of the tool before the GI-2 outcome is met. In this context, a patient will not have experienced resumption of expulsory function, so will not rate it as a symptomatic item. This perhaps supports the use of PRO-diGI after the GI-2 outcome is met. PRO-diGI also provides a wider focus on symptoms important to patients, such as impact on eating, which was highlighted as an important aspect of recovery in qualitative interviews.

The PRO-diGI study has highlighted some challenges for PROM development and application in this field. First, how is it best to recruit people to interviews and surveys close to their acute event? This is important, as temporal distance from an acute experience might lead to issues with recall bias. Second, there are issues around the definition of stability. Stage 1 demonstrated that gastrointestinal recovery is a process that may continue for several weeks. For reasons of pragmatism, this study selected a window around the time of hospital discharge when stability was expected for stage 4a. Identifying and accepting such windows may be necessary for future work.

The development process has not been without limitations. Whilst efforts were made to ensure representation from different ethnic groups who use the healthcare system in each stage, this was not always achieved. At each stage, representation according to sex varied between the majority of participants being female and the majority of participants being male. The median age of participants stayed around 63 years in each stage, which likely reflects the prevalent population undergoing major surgery^14,28^. However, this may mean experiences of younger patients are not fully reflected. At all stages, the percentage of white ethnic participants was >90%, which exceeds the 80% representation in the wider UK population^29^. The research team made significant efforts to encourage teams to ensure representative recruitment from non-white ethnic groups; however, this had limited impact. This may be in part due to the requirement for fluent English in participants to develop a PROM or may reflect the local populations served. There is additional work required for translation into relevant languages, along with cultural adaptation of terminology used for including symptoms and aspects related to the delivery of the instrument. Consideration of site selection is also important for future studies. Notably, those who had not met the GI-2 outcome at day 3 had better scores in the bowel domain than those who had met the outcome. This might reflect them not experiencing incontinence or soiling, as they have not yet resumed bowel function. This has implications for the timing of administration post-surgery. The limited difference in other domains when comparing GI-2 status might reflect small *n* values, meaning retesting of the hypothesis on a larger value is merited. The unexpected performance of scores in relation to GI-2 status might reflect the population recruited, in that patients undergoing planned surgery likely had normal or good function before surgery. Questions are also focused on urgency and incontinence. Patients who have passed GI-2 will be able to answer this, whereas those who have not will be unable. This means that the tool may be best deployed later in recovery rather than in the immediate postoperative phase. The responsiveness analysis is also limited, as no patient anchor was used. Further work is required to explore the impact of factors such as major complications on scores and to calculate a minimum clinically important difference.

There are several strengths for this study. There was extensive patient input throughout, supported by a multidisciplinary team of researchers. This ensures the tool is relevant to patients. During stage 2, experts from outside of the UK, particularly from anglophone countries, were engaged with. This led to edits of the tool to make it relevant to non-UK English speakers, which should improve generalizability and uptake.

Whilst further assessment is required to complete the assessment of the PROM characteristics, researchers could implement this tool into future perioperative trials as a secondary outcome where gastrointestinal dysfunction is anticipated. With the caveats above, policy makers may add this measure to routine data capture around emergency laparotomy, colorectal cancer surgery, and cystectomy.

In summary, PRO-diGI is a potential tool for patients and clinicians to explore and communicate about gastrointestinal recovery after surgery. Once full assessment of responsiveness is complete PRO-diGI can potentially be used in practice. Subsequent implementation into routine practice may help measure quality of care and use in research will provide novel data on treatment efficacy.

## Collaborators

Ewen Griffiths, Rebecca Hancox, Arlo Whitehouse, Michelle Bates, Claire McNeill, Manijeh Ghods, and Andrew McDarby (University Hospitals Birmingham, Birmingham, UK). Andrew Shepperson, Clare Hutton, and Syed Rahman-Casana (Darlington Memorial Hospital, Darlington, UK). Daniel Ashmore, Georgia Hooton, Rebecca Pugh, and Timothy Wilson (Doncaster and Bassetlaw Teaching Hospitals, Doncaster, UK). Victoria Allinson and John Liam O'Hare (Durham Hospital, Durham, UK). Bernadette Collinson and Karen Convery (Norfolk and Norwich University Hospitals, Norwich, UK). James Clark, Charlotte Barker-Kirby, Eve Fletcher, and Suzanne Dean (Royal Cornwall Hospital, Truro, UK). Eleanor Walker, Frank McDermott, Linda Park, Melissa-Rose Bennett, and Sophie Ashman (Royal Devon and Exeter Hospital, Exeter, UK). Sammy Conroy and Caroline Steele (Sheffield Teaching Hospitals, Sheffield, UK). Charmaine Shovelton, Kate James, Eric Mbogu, Karen Roberts, and Lucia Sharp (Musgrove Park Hospital, Taunton, UK). Lynne Palmer, Amy Smith, Fiona Wakinshaw, Jennifer Henderson, and Madeleine Richardson (South Tyneside and Sunderland Hospital, South Shields, UK). Cleo Kenington, Claire Gilmartin, Hong Ju, and Mercedes Lucas Mejia (St George's Hospital, London, UK).

## Supplementary Material

znaf055_Supplementary_Data

## Data Availability

The research team will consider reasonable requests for data sharing of unidentified quantitative data sets for appropriate methodological reasons. The corresponding author should be contacted in the first instance. In addition, the study protocol and other documents, including template consent forms, may be accessed by contacting the corresponding author.
