# Supplementary material for: Development of a patient-reported outcome measure for gastrointestinal recovery after surgery (PRO-diGI)
Source: Br J Surg. 2025 Apr 10;112(4):znaf055. doi: 10.1093/bjs/znaf055 (PMC11983275; doi:10.1093/bjs/znaf055)
Supplement: znaf055_Supplementary_Data [file znaf055_supplementary_data.docx]

**Development of a Patient Reported Outcome measure for GastroIntestinal recovery after surgery (PRO-diGI).**

MJ Lee^1,2^, DM Baker^3^, D Hawkins^4^, S Blackwell^5^, R Arnott^5^, D Harji^6^, G Thorpe^7^, SJ Chapman^8^, GL Jones^9^ on behalf of the PROdiGI Collaborators*

1. Department of Applied Health Sciences, College of Medical and Dental Sciences, University of Birmingham, Birmingham, UK
2. Division of Clinical Medicine, Sheffield Medical School, University of Sheffield, Sheffield, UK
3. Department of Surgery, Leeds Teaching Hospitals NHS Foundation Trust
4. Academic Directorate of General Surgery, Sheffield Teaching Hospitals NHS Foundation Trust, Sheffield, UK
5. Lay representative, Birmingham UK
6. Manchester Foundation NHS Trust, Manchester, UK
7. School of Health Sciences, University of East Anglia, Norwich, UK
8. Leeds Institute of Medical Research at St. James’s, University of Leeds, UK
9. Psychology, School of Humanities and Social Sciences, Leeds Beckett University, Leeds, UK

**Corresponding author.** Mr Matthew Lee, University of Birmingham, [m.j.lee.1@bham.ac.uk](mailto:m.j.lee.1@bham.ac.uk) **ORCID ID**; **0000-0001-9971-1635**

**Supplementary Materials - Index**

| **Supplementary Figures and Tables** |  |
| --- | --- |
| Appendix A: PRO-diGI Collaborators | *pag. 2* |
| Appendix B: COSMIN Taxonomy and development of PRO-diGI | *pag. 3* |
|  |  |

**Supplementary Figures and Tables**

**Appendix A: PROdiGI Collaborators**

| **University Hospitals Birmingham** | Ewen Griffiths; Rebecca Hancox; Arlo Whitehouse; Michelle Bates; Claire McNeill; Manijeh Ghods;Andrew McDarby |
| --- | --- |
| **Darlington Hospital** | Andrew Shepperson; Clare Hutton; Syed Rahman-Casana; |
| **Doncaster and Bassetlaw Teaching Hospitals** | Daniel Ashmore; Georgia Hooton; Rebecca Pugh; Timothy Wilson |
| **Durham Hospital** | Victoria Allinson; John Liam O’Hare |
| **Norfolk and Norwich University Hospital** | Bernadette Collinson; Karen Convery |
| **Royal Cornwall Hospital** | James Clark; Charlotte Barker-Kirby; Eve Fletcher; Suzanne Dean |
| **Royal Devon and Exeter Hospital** | Eleanor Walker; Frank McDermott; Linda Park; Melissa-Rose Bennett; Sophie Ashman |
| **Sheffield Teaching Hospitals** | Sammy Conroy; Caroline Steele; |
| **Musgrove Park Hospital** | Charmaine Shovelton; Kate James; Eric Mbogu; Karen Roberts; Lucia Sharp |
| **South Tyneside and Sunderland NHS Trust** | Lynne Palmer; Amy Smith; Fiona Wakinshaw; Jennifer Henderson; Madeleine Richardson |
| **St Georges Hospital** | Cleo Kenington; Claire Gilmartin; Hong Ju; Mercedes Lucas Mejia |

**Appendix B: COSMIN Taxonomy and Development of PRO-diGI**

| **Domain** | **Sub-domain** | **Phase where assessed** |
| --- | --- | --- |
| **Reliability** | ***Reliability*** | **4a** |
|  | ***Measurement error*** | **4a** |
|  | ***Internal Consistency*** | **3** |
| **Validity** | ***Face Validity*** | **2,4a** |
|  | ***Structural Validity*** | **3** |
|  | ***Hypothesis testing*** | **4b** |
